# Umbilical cord mesenchymal stem cells modulate dextran sulfate sodium induced acute colitis in immunodeficient mice

**DOI:** 10.1186/s13287-015-0073-6

**Published:** 2015-04-16

**Authors:** Antara Banerjee, Debora Bizzaro, Patrizia Burra, Rosa Di Liddo, Surajit Pathak, Diletta Arcidiacono, Andrea Cappon, Patrizio Bo, Maria Teresa Conconi, Marika Crescenzi, Claudia Maria Assunta Pinna, Pier Paolo Parnigotto, Malcolm R Alison, Giacomo Carlo Sturniolo, Renata D’Incà, Francesco Paolo Russo

**Affiliations:** Department of Surgery, Oncology and Gastroenterology, Gastroenterology/Multivisceral Transplant Unit, University Hospital Padova, Via Giustiniani 2, Padova, 35128 Italy; Department of Pharmaceutical and Pharmacological Sciences, University of Padova, Via Marzolo 5, 35131 Padova, Italy; Venetian Institute of Molecular Medicine (VIMM), Via Orus, 2 35129 Padova, Italy; Obstetrics and Gynecology Unit, Cittadella Hospital, via Casa di ricovero, 40 35013 Cittadella Padova, Italy; Centre for Tumour Biology, Barts Cancer Institute, Charterhouse Square, London, EC1M 6BQ UK

## Abstract

**Introduction:**

Inflammatory bowel diseases (IBD) are complex multi-factorial diseases with increasing incidence worldwide but their treatment is far from satisfactory. Unconventional strategies have consequently been investigated, proposing the use of cells as an effective alternative approach to IBD. In the present study we examined the protective potential of exogenously administered human umbilical cord derived mesenchymal stem cells (UCMSCs) against Dextran Sulfate Sodium (DSS) induced acute colitis in immunodeficient NOD.CB_17_-*Prkdc*^scid^/J mice with particular attention to endoplasmic reticulum (ER) stress.

**Methods:**

UCMSCs were injected in NOD.CB_17_-*Prkdc*^scid^/J via the tail vein at day 1 and 4 after DSS administration. To verify attenuation of DSS induced damage by UCMSCs, Disease Activity Index (DAI) and body weight changes was monitored daily. Moreover, colon length, histological changes, myeloperoxidase and catalase activities, metalloproteinase (MMP) 2 and 9 expression and endoplasmic reticulum (ER) stress related proteins were evaluated on day 7.

**Results:**

UCMSCs administration to immunodeficient NOD.CB_17_-*Prkdc*^scid^/J mice after DSS damage significantly reduced DAI (1.45 ± 0.16 vs 2.08 ± 0.18, p < 0.05), attenuating the presence of bloody stools, weight loss, colon shortening (8.95 ± 0.33 cm vs 6.8 ± 0.20 cm, p < 0.01) and histological score (1.97 ± 0.13 vs 3.27 ± 0.13, p < 0.001). Decrease in neutrophil infiltration was evident from lower MPO levels (78.2 ± 9.7 vs 168.9 ± 18.2 U/g, p < 0.01). DSS treatment enhanced MMP2 and MMP9 activities (>3-fold), which were significantly reduced in mice receiving UCMSCs. Moreover, positive modulation in ER stress related proteins was observed after UCMSCs administration.

**Conclusions:**

Our results demonstrated that UCMSCs are able to prevent DSS-induced colitis in immunodeficient mice. Using these mice we demonstrated that our UCMSCs have a direct preventive effect other than the T-cell immunomodulatory properties which are already known. Moreover we demonstrated a key function of MMPs and ER stress in the establishment of colitis suggesting them to be potential therapeutic targets in IBD treatment.

## Introduction

Inflammatory bowel diseases (IBD) are complex multifactorial diseases showing increasing prevalence worldwide [1]. The two principal conditions that involve inflammation of the intestine are ulcerative colitis and Crohn’s disease. Ulcerative colitis involves the large intestine with contiguous inflammation of the colonic lamina propria, accelerated breakdown of extracellular matrix and disruption of the mucosal barrier [2,3], with an excessive production of a variety of inflammatory mediators such as proteolytic enzymes, cytokines, growth factors and reactive oxygen species (ROS) [2,4]. Recent studies link endoplasmic reticulum (ER) stress to the pathogenesis of IBD [5,6]. ER stress arises from conditions that cause the accumulation of misfolded or unfolded proteins within the ER lumen.

ER stress induces activation of the unfolded protein response (UPR), a signaling network that is required to resolve stress, restoring ER homeostasis and promoting cell survival and adaptation. Conversely, under unresolvable ER stress conditions, the UPR promotes inflammation and apoptosis. ER stress has a unique role in the epithelium and can be both a primary cause as well as a consequence of intestinal inflammation. The epithelial cells of the gastrointestinal tract, because of their barrier function, are exposed to toxins and infectious agents that can adversely affect protein folding in the ER and cause ER stress. Recent studies indicate that ER stress could induce inflammation [6-10]. Inflammation can be induced directly by UPR pathways in stressed cells, or indirectly through interaction with immune cells recruited by inflammatory cytokines released from stressed cells [11]. Moreover, reduction of the effectiveness of the mucosal barrier due to decreased secretion of antimicrobial molecules and mucins and premature apoptosis of stressed cells could exacerbate the inflammatory environment typical of IBD.

Despite many advances in basic and clinical science [12,13], treatment of IBD is unsatisfactory. Unconventional strategies have consequently been investigated, identifying the use of stem cells as an alternative approach to treating IBD. Mesenchymal stem cells (MSCs) have emerged as a promising candidate for cellular therapeutics for disorders caused by abnormal immune responses due to their anti-inflammatory and reparative properties [14-21]. Different mechanisms are postulated to be involved in amelioration of colitis by administration of MSCs in animal models and in humans. The principal findings link the amelioration of clinical signs of colitis to local anti-inflammatory actions [19] through suppressed expression of cytokines such as interferon gamma, interleukin-23 and interleukin-17 [21,22].

The present study aimed to demonstrate whether umbilical cord mesenchymal stem cells (UCMSCs) have a direct action other than their T-cell immunomodulatory effect in an experimental model of colitis. For this purpose we induced experimental colitis in immunodeficient mice by administration of dextran sulfate sodium (DSS). The NOD.CB_17_-*Prkdc*^scid^/J mice are characterized by the absence of functional T cells and B cells, have impaired natural killer cell function and have severe combined immunodeficiency. We focused on the reduction in damaged epithelial barrier integrity, the reduction in inflammatory infiltration and the modulation of the UPR.

## Methods

### Umbilical cord mesenchymal stem cells isolation and expansion

Umbilical cord (UC) collection and processing were approved by the Veneto Region Review Board (4087/03). UC samples were obtained immediately after full-term deliveries, after written informed consent from the mothers at the Obstetrics and Gynaecology Unit of Cittadella Hospital, Padova, Italy, and processed within 24 hours. UCMSCs were isolated and expanded according to the protocol reported previously by our group [14,23]. Briefly, the umbilical vessels were manually removed and the jelly was minced to obtain small fragments; these were cultured in standard medium (Dulbecco’s modified Eagle’s medium high glucose, fetal bovine serum 20%, 1% glutamine, 1% penicillin/streptomycin). UCMSCs started to migrate from the explants within 10 to 15 days.

### *In vitro* characterization of UCMSCs

Cells were analyzed for UCMSCs surface marker expression using anti-human antibodies raised against CD73, CD90, CD166, CD105, CD44, integrin β1 (CD29), c-kit and human leukocyte antigens-DR (all from BD Biosciences, San Diego, CA, USA). As negative controls, isotype antibodies conjugated with fluorescein isothiocyanate and R-phycoerythrin (Santa Cruz Biotechnology, Santa Cruz, CA, USA) were used. The cytofluorimetric analyses were performed with the MoFlo High-Speed Cell Sorter (DAKO-Beckman Coulter, Carpinteria, CA, USA) and data were analyzed using Summit 4.3 software (DAKO-Beckman Coulter).

Mesenchymal stem cell features of UCMSCs were investigated by adipogenic and osteogenic differentiation assays and their fetal origin was verified through sex-determining region Y gene analysis, as described previously by our group [23].

### Induction of dextran sulfate sodium colitis in NOD.CB_17_-*Prkdc*^scid^/J mice

Twenty healthy NOD.CB_17_-*Prkdc*^scid^/J male mice, 7 to 9 weeks old and weighing 18 to 25 g, were obtained from Charles River Laboratories (Wilmington, MA, USA). The mice were maintained in a pathogen-free room, housed individually and fed with an autoclaved pellet diet and water *ad libitum*. All experiments were conducted in strict accordance with the institutional guidelines for animal research and approved by the Directorate-General for Animal Health and Veterinary Drugs of the Italian Ministry of Health in accordance with the law on animal experimentation (DL 116/92, protocol number 76/2010/B, approval date 7 April 2010). Furthermore, all animal treatments were reviewed and approved in advance by the ethics committee of the University of Padova, Italy.

Experimental acute colitis was induced in mice by oral administration of 3.5% (wt/vol) DSS (TdB Consultancy AB, Uppsala, Sweden) in sterile drinking water for 7 days. The DSS (molecular weight ~40 kDa) solution was prepared fresh daily. At day 1 after commencement of DSS treatment, mice were randomly divided into two groups (*n* = 5) and injected intravenously with either 1 × 10^6^ cells in 100 μl phosphate-buffered saline (PBS) per animal (DSS + UCMSCs group) or with 100 μl PBS alone (DSS group). Healthy mice injected with UCMSCs via the tail vein per time (UCMSCs group, *n* = 5) and healthy mice fed with a normal diet and sterile water (negative control group, *n* = 5) were used as controls.

### Assessment of the severity of colitis and histological examination

To examine the severity of colitis, stool consistency, fecal bleeding and weight loss were each evaluated daily on a 0 to 4 point scale and averaged for an overall disease activity index (DAI). On day 7, mice were anesthetized by inhalation of isofluorane, blood samples were collected by cardiac puncture and animals were then sacrificed by cervical dislocation. The entire colon was excised and carefully washed in PBS. Colon length was measured before sectioning for specific analysis. Colonic inflammation and damage were analyzed by histological examination. Sections of the distal colon 1 cm long were cut out longitudinally and fixed in 10% formalin for at least 24 hours and then embedded in paraffin wax for histological analysis. Distal colon sections 4 μm thick were stained with hematoxylin and eosin to address the degree of inflammation. The damage was scored blindly, with slight modification, as reported by Iba and colleagues [24].

Staining of goblet cells and assessment of the collagen deposition were performed by periodic acid–Schiff and Masson’s trichrome staining respectively according to the manufacturer’s protocol (Sigma Aldrich, Saint Louis, MO, USA).

### Scores for disease activity index calculation

The weight loss percentage was scored as: grade 0, none; grade 1, 1 to 5%; grade 2, 5 to 10%; grade 3, 10 to 20%; grade 4, >20%. Fecal bleeding was scored as grade 0, no bleeding; score 1, few blood-tinged stools; score 2, some bleeding; grade 3, gross bleeding; grade 4, blood filling the whole colon. The score for stool consistency was: grade 0, normal stool; grade 1, slightly loose stool; grade 2, loose stools; grade 3, watery stool; score 4, severe diarrhea (according to Cooper and colleagues [25], with slight modifications).

### Scores for histological analysis

The score for histological analysis was as follows: loss of epithelium (0 = none; 1 = 0 to 5%, mild; 2 = 5 to 10%, moderate; 3 = >10%, severe): crypt damage as percentage loss of crypt (0 = none; 1 = 0 to 10%, mild; 2 = 10 to 20%, moderate; 3= >20%, severe): depletion of goblet cells (0 = none; 1 = mild; 2 = moderate; 3 = severe): and infiltration of inflammatory cells (0 = none; 1 = mild; 2 = moderate; 3 = severe).

### Localization of exogenously administered UCMSCs

Sections were stained with a standard immunohistochemistry procedure with specific Anti-Human Nuclear Antibody (MAB1281; Chemicon, Millipore Corporation, Billerica, MA, USA) to identify the localization of exogenously administered UCMSCs in the colonic tissues. No reactivity against mouse antigens was guaranteed by the manufacturer.

### Tissue myeloperoxidase assay and serum catalase assay

Myeloperoxidase (MPO) activity, an index of the inflammatory response, was assayed in colonic tissues as described previously by Islam and colleagues [26]. Catalase activity as an index of oxidative stress was measured on serum samples employing the method of Chance and Maehly [27]. The catalase activity was expressed as units per milligram of protein, and units of enzyme activity is defined as the amount of enzyme required to degrade 1 μmol H_2_O_2_ per second per milligram of protein.

### Matrix metalloproteinase activity

Expression of matrix metalloproteinase (MMP)2 and MMP9, which are well known to be involved in intestinal disorders and IBD, was evaluated. Colonic tissues were washed with cold PBS and tissues were lysed by lysis buffer (0.05 M Tris, 0.2 M NaCl, 0.010 M CaCl_2_, 0.5% Triton X-100) for 20 minutes on ice. Cellular debris was removed by centrifugation and protein concentration was estimated using a BCA™ protein assay kit (Pierce Diagnostics, Rockford, IL, USA) on the supernatant. Tissue lysates were subjected to gelatin zymography [28].

### Western blot analysis of endoplasmic reticulum stress markers

Colonic tissues were lysed in extraction buffer (100 mM KCl, 3 mM NaCl, 3.5 mM MgCl_2_, and 10 mM HEPES; pH 7.4) containing 1% Triton X-100, 1× Protease Inhibitor Cocktail (Calbiochem, Milan, Italy), and were centrifuged (1,500 × *g* for 30 minutes at 4°C). Protein concentration was determined in the supernatant using the BCA™ protein assay kit (Pierce Diagnostics). SDS-PAGE and transfer to nitrocellulose membranes was performed using standard procedures. Membranes were analyzed for the expression of specific markers of ER stress activation: the ER stress chaperone binding immunoglobulin protein (BiP), the ER stress sensor PKR-like endoplasmic reticulum kinase (PERK) and the protein disulfide isomerases (PDI) according to the manufacturer’s protocol (Cell Signaling Technology, Inc., Danvers, MA, USA). Images were acquired and digitally scored with a densitometer image analyzer (Quantity one; Bio Rad, Hercules, CA, USA).

### Statistical analyses

Data are presented as the mean ± standard deviation. Student’s *t* test was used to assess differences between groups. *P* <0.05 was assumed to indicate a significant difference. Data analyses were performed with SPSS (IBM Corp., Armonk, NY, USA) and StatsDirect (Altrincham, UK).

## Results

### Umbilical cord mesenchymal stem cells isolation and phenotype analysis

**Figure 1:**
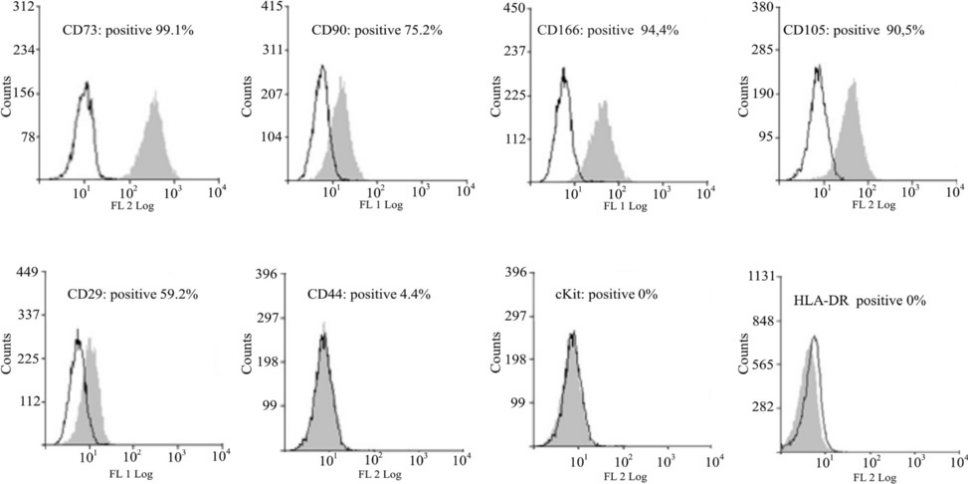
Cellular characterization of umbilical cord mesenchymal stem cells. Flow cytometry analysis of umbilical cord mesenchymal stem cells showed a mesenchymal phenotype. Cells were positive for typical mesenchymal markers (CD29, CD73, CD90, CD105 and CD166) while hematopoietic markers (CD44 and c-kit) were weakly or not expressed. Human leukocyte antigen-DR (HLA-DR) was not expressed at all.

UCMSCs started to migrate from the explants within 10 to 15 days and gave rise to a homogeneous population of adherent spindle-shaped cells with a fibroblastic morphology. These cells fulfill the minimal criteria for defining MSCs as reported by the International Society for Cellular Therapy [29]. Indeed, in agreement with our previous studies [14,23], flow cytometry analysis showed high expression of typical mesenchymal cell markers such as CD166, CD105, CD90, CD73 and CD29. Hematopoietic and endothelial markers CD44 and c-kit were weakly or not expressed and human leukocyte antigen-DR was not expressed at all (Figure 1). Moreover, UCMSCs were able to differentiate toward adipogenic and osteogenic lineages (as reported previously by our group [23]).

### Umbilical cord mesenchymal stem cells reduce disease severity in DSS-induced colitis

In NOD.CB_17_-*Prkdc*^scid^/J mice, administration of 3.5% DSS for 7 days induced severe colitis localized mainly in the distal colon. All DSS-treated mice developed disease symptoms similar to colitis in humans, including body weight loss, bloody diarrhea and shortening of the colon with a progressive increase of the DAI, which included scores of weight loss, stool consistency and fecal bleeding.

**Figure 2:**
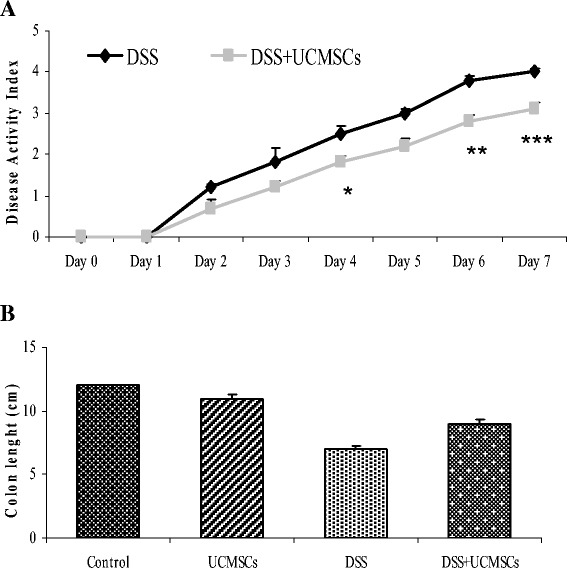
Clinical and therapeutic efficacy of exogenous umbilical cord mesenchymal stem cell administration. **(A)** Disease activity index (DAI). In the umbilical cord mesenchymal stem cells (UCMSCs)-treated group, the DAI score was significantly downregulated on days 4, 6 and 7 of treatment. **(B)** Colon length. UCMSCs in dextran sulfate sodium (DSS)-treated mice were able to reduce the degree of DSS-induced colon shortening compared with the DSS + phosphate-buffered saline group. Values reported as mean ± standard deviation. *P <0.05, **P <0.01, ***P <0.001.

UCMSCs administration at days 1 and 4 in DSS-treated mice improved all of the disease parameters. DAI scores, calculated daily, were lower in these mice compared with those treated with DSS alone throughout the 7 days of observation and these reductions were significant at days 4, 6 and 7 (*P* <0.05 at day 4, *P* <0.01 at day 6 and *P* <0.0001 at day 7) (Figure 2A).

Moreover, DSS mice treated with UCMSCs did not suffer the same degree of DSS-induced colon shortening as the DSS + PBS group (8.95 ± 0.33 cm vs. 6.8 ± 0.20 cm respectively, *P* <0.01; Figure 2B). Control group mice did not show any signs of colitis and gained weight over time.

**Figure 3:**
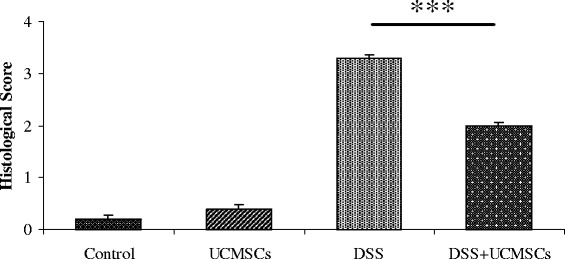
Therapeutic efficacy of umbilical cord mesenchymal stem cell treatment on the histological colitis score. Administration of umbilical cord mesenchymal stem cells (UCMSCs) in dextran sulfate sodium (DSS)-treated mice significantly improved histological scores. Values reported as mean ± standard deviation. ***P <0.001.

**Figure 4:**
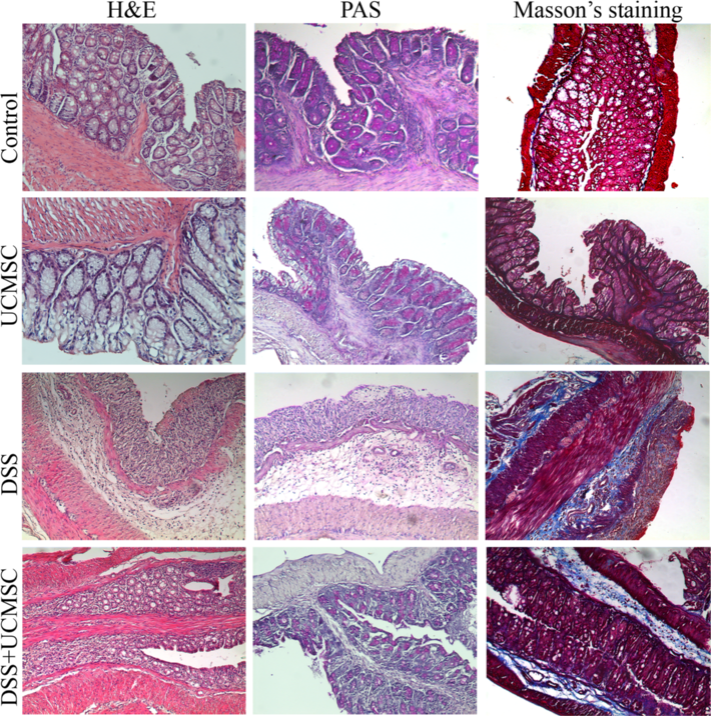
Histological analysis of the distal colon on day 7 of control mice (no dextran sulfate sodium (DSS)), umbilical cord mesenchymal stem cell**-**treated mice, DSS + phosphate-buffered saline-treated mice and DSS + umbilical cord mesenchymal stem cell-treated mice. Hematoxylin and eosin (H&E) staining showed improvement after umbilical cord mesenchymal stem cell (UCMSCs) treatment because it reduced the extent of the inflamed area, crypt damage, edema of submucosa and infiltration of inflammatory cells. Periodic acid–Schiff (PAS) staining detected goblet cells that displayed a strong purple/magenta color when stained with PAS reagent. There was complete depletion of goblet cells in the crypts of the dextran sulfate sodium (DSS) + phosphate-buffered saline mice. In contrast, DSS + UCMSCs mice showed retention of some goblet cells. Masson’s trichrome staining showed increased deposition of collagen after DSS treatment (blue/green-stained areas represent collagen deposits).

Colitic mice treated with DSS showed disordered mucosal architecture with loss of crypts, diffuse depletion of goblet cells, inflammatory cell infiltration, edema and epithelial cell necrosis. In contrast, the colonic tissue from DSS + UCMSCs-treated animals showed a significant reduction of histological score (1.97 ± 0.13 vs. 3.27 ± 0.13, *P* <0.001; Figure 3) with only focal depletion of goblet cells, and less inflammatory cell infiltration within the lamina propria (Figure 4).

To investigate whether the marked rigidity found in the distal colon of DSS-treated mice was due to inflammation/infiltration or fibrosis, Masson’s trichrome staining for collagen deposition was performed.

Collagen deposition, evident as blue/green color staining in the mucosa and submucosa, was more marked in DSS mice compared with controls, presumably contributing to the increased rigidity of the inflamed region. In contrast, the degree of collagen deposition was greatly reduced in both mucosa and submucosa of the DSS + UCMSCs group (Figure 4).

### Umbilical cord mesenchymal stem cells localize to the colon of DSS-treated mice

**Figure 5:**
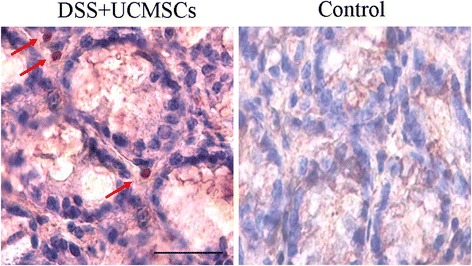
Localization of exogenously administered umbilical cord mesenchymal stem cells. Immunohistochemical analysis of the inflamed colon in umbilical cord mesenchymal stem cell (UCMSCs)-treated mice compared with control mice. Human nuclei of UCMSCs were observed in the lamina propria of colon of dextran sulfate sodium (DSS)-treated mice (red arrows), whereas no such human nuclei were observed in the control mice.

To study whether UCMSCs could be found in the DSS-damaged colon, immunohistochemical analysis for human nuclei was performed. UCMSCs were detected in the inflamed distal colon of colitic mice (Figure 5), but not in the colons of healthy mice infused with UCMSCs (Figure 5).

### Umbilical cord mesenchymal stem cells reduce inflammatory tissue infiltration but do not influence antioxidant activity

**Figure 6:**
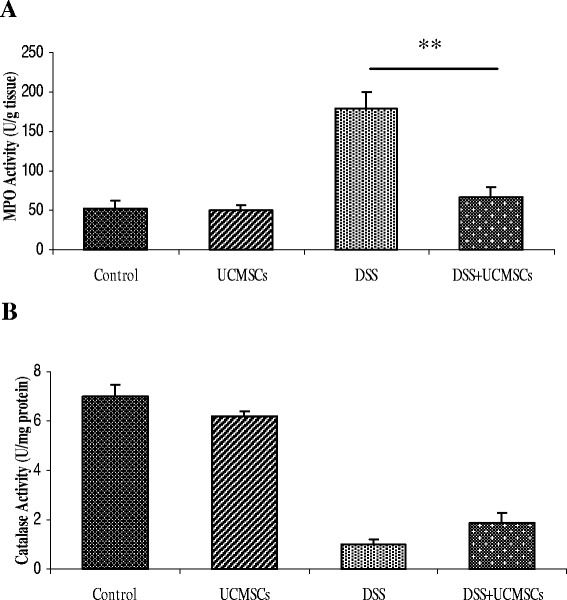
Cytotoxicity study of myeloperoxidase activity in the colon and catalase activity in serum. Cytotoxicity studies were performed by measuring **(A)** myeloperoxidase activity in the colon and **(B)** catalase activity in serum. Mice receiving dextran sulfate sodium (DSS) + umbilical cord mesenchymal stem cell (UCMSCs) treatment showed a significantly reduced myeloperoxidase activity compared with the DSS + phosphate-buffered saline (PBS) group (**P <0.01). On the other hand, DSS + UCMSCs-treated mice had almost similar catalase levels to the DSS + PBS mice.

MPO activity was used as a measure of inflammation induced by innate immunity, and was found to be significantly increased (*P* <0.001) by DSS treatment at day 7 (168.95 ± 18.22 U/g vs. 63.46 ± 13.46 U/g in control mice). Compared with the DSS colitic mice, the MPO activity in the colon of DSS + UCMSCs animals was significantly lower (78.23 ± 9.71 U/g vs. 168.95 ± 18.22 U/g, *P* <0.01; Figure 6A). These data suggest that the UCMSCs infusion inhibits DSS-induced tissue inflammatory infiltration.

To estimate the level of induced oxidative stress, catalase activity was measured in the serum. A significant reduction in the activity of catalase was observed in the colon of DSS-treated mice compared with controls, indicating the exhaustion of antioxidant enzymes. Administration of UCMSCs in DSS-treated mice failed to maintain the initial level of catalase activity (1.53 ± 0.20 vs. 1.19 ± 0.07 in DSS mice; Figure 6B).

### MMP2 and MMP9 expression in the colon

**Figure 7:**
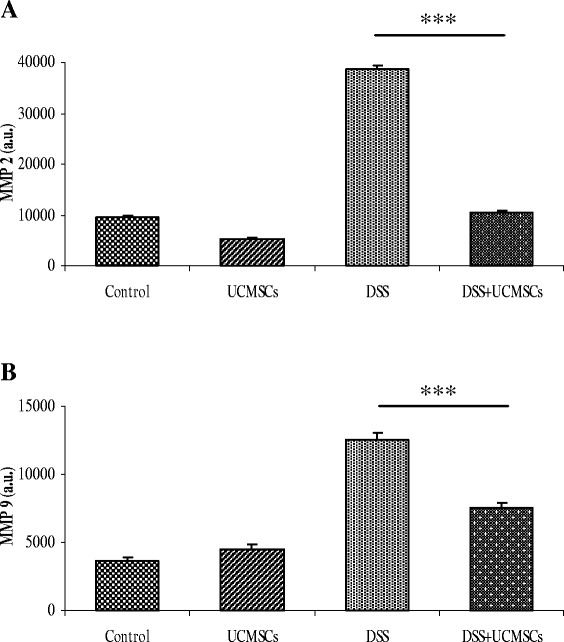
Gelatin zymograms (7.5% SDS-PAGE) showing matrix metalloproteinase activity in the colon. Matrix metalloproteinase MMP2 **(A)** and MMP9 **(B)** activity in the colon. The densitometric intensity of bands is shown in the bar graphs. There was elevated expression of both MMP2 and MMP9 in the dextran sulfate sodium (DSS) + phosphate-buffered saline mice compared with controls, and there was significantly less expression in the DSS + umbilical cord mesenchymal stem cells (UCMSCs) mice (***P <0.001). Values are mean ± standard deviation of three independent experiments. a.u., arbitrary units.

MMPs are extracellular proteinases with proteolytic activity against extracellular matrix proteins. We examined MMP2 and MMP9 activities that are reported to be involved in IBDs. Colons of control mice showed low MMP2 and MMP9 activity as measured by gelatin zymography. Treatment with DSS induced a significant upregulation in activities of both MMPs, while activities in DSS + UCMSCs animals were significantly lower, at levels comparable with controls (*P* <0.001) (Figure 7).

### Umbilical cord mesenchymal stem cells modulate the expression of endoplasmic reticulum stress proteins

**Figure 8:**
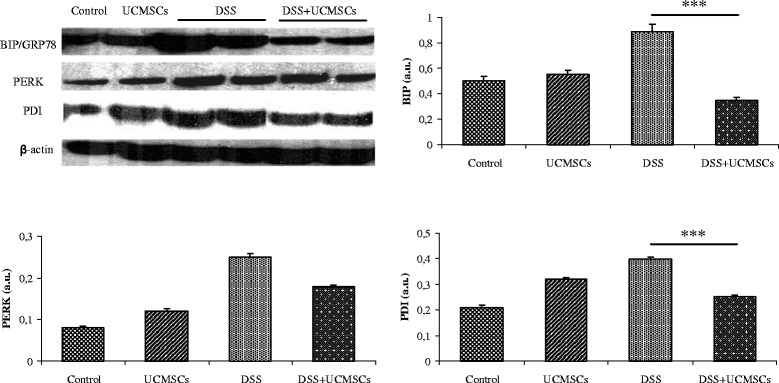
Western blot analysis of the endoplasmic reticulum stress response in the colon of control and treated mice. The densitometric analysis showed a significant decrease in binding immunoglobulin protein (BIP) and protein disulfide isomerases (PDI) in the dextran sulfate sodium (DSS) + umbilical cord mesenchymal stem cells (UCMSCs) mice when compared with the DSS + phosphate-buffered saline mice. (***P <0.001). β-actin was used as a loading control. The densitometric intensity of bands is shown in the bar graphs. Values are mean ± standard deviation of three independent experiments. a.u., arbitrary units; PERK, PKR-like endoplasmic reticulum kinase.

Given the evidence for a role of ER stress proteins in intestinal diseases, we measured the expression of specific proteins of the UPR pathway. To study the three principal phases of UPR (initiation, signal transduction and downstream effect) we measured expression of: BiP, a chaperone that has been extensively used as biological marker for onset of the UPR; the major transducer of the ER stress response protein kinase PERK; and the UPR-induced foldase PDI. Analysis of these markers showed a significant induction in the distal colon that coincided with the clinical and histological signs of DSS-induced colitis. Administration of UCMSCs induced a significant reduction in the amount of BIP and PDI that was comparable with controls (*P* <0.001). On the contrary, PERK expression was not affected by UCMSCs administration (Figure 8).

## Discussion

Cellular therapy with stem cells and their progeny is a promising new approach capable of addressing as yet unmet medical needs in various inflammatory and autoimmune diseases [30]. Recent studies provide ample evidence that MSCs can promote regeneration of wounded tissues, modulate the systemic immune system and have anti-inflammatory properties [22,31-33]. Our present and past data clearly demonstrate that human UC from full-term deliveries can be successfully used as a source of MSCs due to their ease of isolation and reproducibility to obtain significant numbers of cells. UCMSCs are plastic adherent and highly proliferative cells, and also express a panel of surface markers that are in line with proposed minimal criteria set by the International Society for Cellular Therapy [29].

The beneficial effects of MSCs have been mostly attributed to paracrine effects, the release of signaling factors for tissue repair, rather than transdifferentiation or fusion with cells in injured areas [34]. These paracrine effects have been attributed to immunosuppressive cytokines that modulate immune responses by inhibiting the effector T-cell responses and by increasing the number of T-regulatory cells [22,35]. Currently, the precise mechanisms by which MSCs act remain unclear, but MSCs seem to have effects at multiple levels, not just in a single immune response pathway.

In the present study we aimed to assess whether UCMSCs have a direct therapeutic effect other than their T-cell immunomodulatory effect. We evaluated the effects of exogenously administered UCMSCs in DSS-induced acute colitis in an experimental mouse model of immunodeficiency. In this way we aimed to exclude the previously assessed T-cell immunomodulatory contribution of MSCs in chemically induced colitis in mice. To this end we used NOD.CB_17_-*Prkdc*^scid^/J mice, which are characterized by absent functional T cells and B cells, have impaired natural killer cell function and have severe combined immunodeficiency. Our results are in line with previous reports which have shown that DSS induces colitis in immunodeficient mice independently of T lymphocytes and B lymphocytes [36,37]. DSS administered orally is the cause of epithelial cell toxicity, increased intestinal permeability and macrophage activation. Moreover, recent studies demonstrated by selective knockout of a specific UPR protein that ER stress is a critical player in DSS-induced colitis [38-40]. These elegant studies demonstrated by selective knockout of a specific gene of the UPR pathway that ER stress is a major component of the cascade of events that induce colitis in DSS-treated mice. Taken together these events induce the deleterious effects of DSS, although the specific mechanisms are not fully understood.

We demonstrated that the dose selected (3.5% DSS 40 kDa for 7 days) was capable of inducing acute colitis in immunodeficient NOD.CB_17_-*Prkdc*^scid^/J mice with definitive clinical signs such as diarrhea, gross rectal bleeding and loss of body weight. The lesions induced by DSS were characterized by crypt distortion or entire crypt loss by day 7, complete loss of goblet cells and collagen deposition. The damage compromised normal colonic function and led to loss of body weight and an increase in the DAI score.

In this study the systemic administration of UCMSCs at day 1 and day 4 led to a significant reduction of disease activity, apparent from day 2, and to an amelioration of the histological score with reduction both in submucosa edema and collagen deposition. The fact that this significant amelioration occurred in such a short time frame suggests that UCMSCs have beneficial effects through paracrine activity, rather than through cell differentiation or cell fusion. This hypothesis is supported by the very rapid cell turnover in the intestine.

Interestingly, an increased incidence of goblet cells, which is generally associated with regeneration during the recovery phase of DSS colitis, was also observed at day 7 in those mice also treated with UCMSCs. This might be an indication of less damage resulting in a more normal transit time from the stem cell zone to the surface. On the contrary, at same time point, goblet cells in mice treated with only DSS were diffusely depleted, probably because of the accelerated turnover of the regenerating crypts resulting in insufficient time for goblet cell differentiation.

Several studies have shown a correlation between an increase in ROS production and disease activity in inflamed biopsies of IBD patients [41,42]. Recent studies have linked intestinal oxidative stress to epithelial damage. ROS are generated inside the intestinal tract during the oxidative burst by activated phagocytic cells which possess ROS-producing enzymes such as nicotinamide adenine dinucleotide phosphate hydrate oxidase, nitric oxide synthase and MPO [43].

In the present study we focused our attention on MPO, an enzyme found predominantly in neutrophils and which is a good marker of inflammatory cell infiltration and tissue injury. Indeed, neutrophil infiltration into the inflamed mucosa is one of the most prominent histological features observed in IBD [44]. From our results, the decrease of MPO activity after UCMSCs administration can be explained by a reduction of DSS-induced neutrophil accumulation in inflamed tissue.

Antioxidant enzymes, including catalase, represent the first line of defense against free radicals, and therefore their regulation depends mainly upon the oxidant status of the tissue. In line with the enhanced activity of MPO in DSS-treated mice, we showed a significant reduction in catalase activity compared with control groups, indicating their likely saturation to block DSS-induced massive free radical production. However, UCMSCs in DSS colitis did not influence the activity of catalase, which was similar to that for the DSS-treated mice.

MMPs are a family of proteases involved in turnover of extracellular matrix and cell migration and they have been implicated as one of the main factors involved in the process of tissue destruction and remodeling as well as inflammation in IBD [3,45]. Indeed, MMPs could be released from inflamed intestinal epithelial cells with subsequent loss of mucosal integrity, thus facilitating penetration of inflammatory cells into the colon. An imbalance between degradative and reparative processes of the extracellular matrix can induce crypt damage [46]. While the role of MMPs in pathogenesis of IBD is well known, which MMPs are involved is still controversial. Heimesaat and colleagues demonstrated that MMP2 and MMP9 are involved in DSS-induced colitis but while MMP2 is crucial in the pathogenesis of colitis, MMP9 seems not to be essential [47]. On the other hand, Castaneda and colleagues demonstrated that MMP-9^−/−^ mice exposed to DSS or salmonella had a significantly reduced severity of colitis [48]. However, other groups have demonstrated that other forms of MMPs alter the nature of DSS-induced colitis. For example Park and colleagues indicated that MMP3 and MMP9 are the principal MMPs [49], while Koelink and colleagues ascribed the crucial role to MMP8 and MMP9 [50]. Given the heterogeneity of MMPs, we focused on the expression of the two most studied metalloproteinases: MMP2 and MMP9. During IBD, MMP2 is highly upregulated and localizes to the subepithelial and pericryptal fibroblasts/myofibroblasts, mononuclear cells (macrophages and lymphocytes), epithelial cells and vascular endothelial cells, while MMP9 is expressed by immune cells (neutrophils, macrophages, lymphocytes) as well as by epithelial cells during inflammation [51]. Our zymograms illustrated that MMP2 and MMP9 were active in the colonic homogenates from the DSS-treated mice. UCMSCs transplantation was able to reduce or block expression of both MMPs, thereby decreasing the protease burden at the site of inflammation and thus maintaining the mucosal integrity, preventing tissue degradation and migration of inflammatory cells into the colon. A possible explanation for this comes from the study of Lozito and Tuan [52], which demonstrated that MSCs caused inhibition of MMPs by secreting high levels of the MMP endogenous inhibitors, the tissue inhibitors of metalloproteinases.

We then focused on determining whether UCMSCs administration could affect the UPR due to ER stress induced by DSS. As reported above, ER stress is crucial in establishment of DSS-induced colitis [38], but the mechanism by which DSS induces ER stress is not known. It was proposed that DSS alters the expression of tight junctions and induces increased epithelial apoptosis and the imbalance between apoptosis and proliferation causes relevant leaks in the epithelial barrier, with consequently inflammation and ER stress [53]. Bertolotti and colleagues also suggested that DSS acts on the epithelial cells which are the first to be exposed to the luminal contents, establishing a cascade of processes that leads to inflammation and ulceration [40]. The observation that the onset of inflammation precedes the development of ulcerative lesions [54,55] suggests that the direct toxicity of DSS resulting in death of epithelial cells is unlikely to account for the initiation of disease. The authors suggested that a DSS-induced perturbation in ER function (ER stress) plays an early role in the development of inflammation and subsequent ulceration. Given these findings, we evaluated protein expression of three mediators of the UPR: BiP, a chaperone that has been extensively used as biological marker for onset of the UPR; PERK, the major transducer of the ER stress response; and a UPR-induced foldase PDI. Expression of all markers was greatly increased in DSS colitic mice compared with control mice. After administration of UCMSCs we observed a significant reduction in the expression of BIP and PDI that reached a level comparable with control mice, but PERK expression was not affected by UCMSCs administration. The cytoplasmic portion of PERK contains a protein kinase domain, which undergoes activating transautophosphorylation by oligomerization in ER-stressed cells. Unfortunately, the antibody we used for our western blot analysis did not distinguish between PERK in its unphosphorylated inactive form and the phosphorylated active form. Similar expression of PERK in DSS colitic mice and UCMSCs + DSS mice could therefore be due to this lack of discrimination between the two forms.

## Conclusions

**Figure 9:**
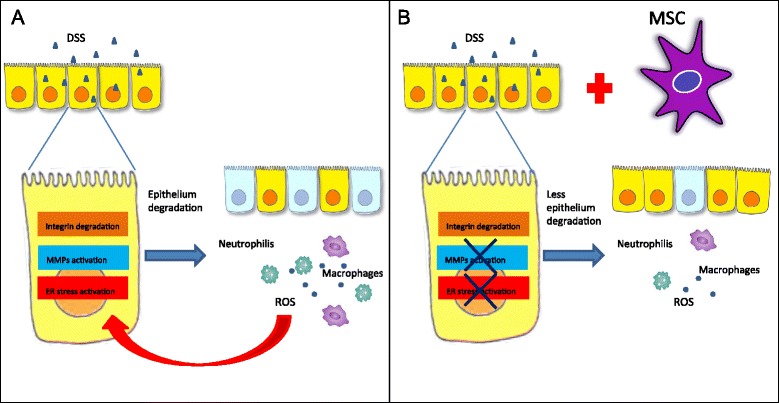
Action of dextran sulfate sodium in immunodeficient mice and role of administration of umbilical cord mesenchymal stem cells in colitic mice. **(A)** Mechanism of action of DSS in immunodeficient mice and **(B)** role of administration of umbilical cord mesenchymal stem cells (UCMSCs) in colitic mice. Dextran sulfate sodium **(**DSS) administration in immunodeficient mice induces integrin degradation, matrix metalloproteinase (MMP) activation and endoplasmic reticulum (ER) stress. These events induce increased epithelial apoptosis and the imbalance between apoptosis and proliferation, causing relevant leaks in the epithelial barrier, with consequent inflammation. Infiltrating neutrophils and macrophages produce reactive oxygen species (ROS) that lead to cell death and further tissue damage. Moreover, ROS is one of the important stimuli that triggers ER stress. All of these events involving MMP activation and ER stress presumably lead to the establishment of colitis with clinical signs comparable with inflammatory bowel diseases in humans, but without the participation of T lymphocytes, B lymphocytes and natural killer cells. UCMSCs administration in colitic mice is able to reduce MMP and ER activation, resulting in less epithelial degradation and inflammation. Consequently, ROS production is lower and the vicious cycle of epithelial damage is inhibited. In this way, UCMSCs are able to prevent DSS-induced colitis. MSC, mesenchymal stem cell.

Our results confirm that NOD.CB_17_-*Prkdc*^scid^/J mice are susceptible to acute DSS colitis, suggesting that T cells, B cells and natural killer cells are not required for disease onset, and that the acute disease may be driven more by neutrophils or other innate immune cell types, in line with previous reports [36,37]. Interestingly we found a vicious cycle induced by DSS administration in immunodeficient mice that included MMP activation, neutrophil infiltration, ROS production and ER stress. Neutrophils are one of the major cells responsible for ROS production and it is known that excessive ROS initiates perturbation of the cellular redox balance leading to cell death and further tissue damage [56]. Moreover, ROS are one of the important stimuli that trigger ER stress [57] and ER stress is often accompanied by increased ROS generation [58] in the so-called paradigm ROS-dependent ER stress. All of these events involving MMP activation and ER stress presumably lead to the establishment of colitis with clinical signs comparable with IBD in humans, but without the participation of T lymphocytes, B lymphocytes and natural killer cells (Figure 9A). On the basis of these results, MMP and ER stress inhibitors could be considered as potential therapeutic targets in IBD treatment.

Our study demonstrated that the systemic infusion of UCMSCs successfully ameliorated the clinical and histological signs of DSS-induced colitis. The reduction of the DAI within 2 days of DSS treatment suggests that the UCMSCs prevent the development of colitic damage through a paracrine mechanism.

In conclusion, our results demonstrate that UCMSCs are able to prevent DSS-induced colitis in immunodeficient mice. Using these mice we demonstrated that our UCMSCs have a direct preventative effect other than through T-cell immunomodulation (Figure 9B). Moreover, our results strongly implicate MMPs and ER stress in the establishment of colitis, suggesting them to be potential therapeutic targets for IBD treatment.

## Abbreviations

BiP: binding immunoglobulin protein
DAI: disease activity index
DSS: dextran sulfate sodium
ER: endoplasmic reticulum
IBD: inflammatory bowel diseases
MMP: matrix metalloproteinase
MPO: myeloperoxidase
MSC: mesenchymal stem cell
PBS: phosphate-buffered saline
PDI: protein disulfide isomerases
PERK: PKR-like endoplasmic reticulum kinase
ROS: reactive oxygen species
UC: umbilical cord
UCMSCs: umbilical cord mesenchymal stem cells
UPR: unfolded protein response
